# Lantadene A and boswellic acid isolated from the leaves of *Lantana camara* L. have the potential to control phytopathogenic *Fusarium* species

**DOI:** 10.1016/j.heliyon.2022.e12216

**Published:** 2022-12-15

**Authors:** Hlabana Alfred Seepe, Lerato Raphoko, Stephen O. Amoo, Winston Nxumalo

**Affiliations:** a Agricultural Research Council—Vegetables, Industrial and Medicinal Plants, Roodeplaat, Private Bag X293, Pretoria 0001, South Africa; b Department of Chemistry, University of Limpopo, Private Bag X1106, Sovenga, 0727, Polokwane, South Africa; c Döhne Agricultural Development Institute, Plant and Crops Production Research, Private Bag X 15, Stutterheim, 4930, South Africa; d Indigenous Knowledge Systems Centre, Faculty of Natural and Agricultural Sciences, North-West University, Private Bag X2046, Mmabatho 2735, South Africa; e Department of Botany and Plant Biotechnology, Faculty of Science, University of Johannesburg, P.O. Box 524, Auckland Park 2006, South Africa

**Keywords:** Antifungal activity, Antimicrobial, Boswellic acid, Biopesticide, *Fusarium* species, Natural fungicide, Phytotoxic

## Abstract

Phytopathogenic *Fusarium* species are restricting factors causing diseases and yield loss in crop production. As part of exploration for pesticides from medicinal plants, this study aimed to isolate and characterize bioactive compounds from *Lantana camara* L. and evaluate their efficiency against *Fusarium* phytopathogens. Phytochemical investigation of ethyl acetate leaf extract led to separation of lantadene A (22-angeloyloxy-9-hydroxy-3-oxo-olean-12-en-28-oic acid) and boswellic acid (11-keto-β-boswellic acid). The chemical structures of the aforementioned compounds were confirmed using physical properties, spectroscopic analysis, and published data. Lantadene A exhibited significant antifungal activity against *F. subglutinans*, *F. proliferatum*, *F. solani*, *F. graminearum*, and *F. semitectum* with minimum inhibitory concentration (MIC) less than or equal to 0.63 mg/mL. Boswellic acid exhibited strong activity (MIC = 0.63 mg/mL) against *F. subglutinans* and *F. semitectum*. In terms of their toxicity towards Raw 264.7 cells, lantadene A and boswellic acid recorded half-maximal inhibitory concentration values of 84.2 μg/mL and 186.6 μg/mL, respectively. Both lantadene A and boswellic acid had no phytotoxic effect against seed germination and seedling root length. Lantadene A and boswellic acid have strong potential to be further investigated as lead natural fungicides (biopesticides) to control *Fusarium* crop diseases.

## 1 Introduction

Many phytopathogenic fungi infect different crops and cause great yield loss. As an example, fungal pathogens including *Fusarium* species can cause about 50%–80% damage to maize during storage [1, 2]. *Fusarium* pathogens can cause diseases in crops such as wheat, maize, potatoes, beans, sorghum, sugar cane and tomatoes [3]. These pathogens can also cause diseases in the field by infecting the roots, stem, leaves, and fruit, and eventually reduce the quality of the crops [4, 5, 6]. The head or seed blights, stem, ear, and root rots, and vascular wilt are among major *Fusarium* crop diseases having great impact on food security in many parts of the world [7, 8]. The economic damage of *Fusarium* pathogens is also through the production of allergenic compounds and mycotoxins, which contaminate fruits, seeds and other agricultural commodities [9, 10]. These mycotoxins are harmful to humans and livestock, and they have a negative effect on food safety and international trade [11, 12]. A successful approach to prevent and control crop diseases is through integrated control management strategies, which may include the application of fungicides [13]. Excessive or inappropriate application of these chemicals leads to increased human health risks, environmental pollution, and the development of resistant fungal strains [14, 15, 16, 17, 18, 19, 20]. In the light of these challenges, the development of eco-friendly and effective alternative fungicides is gaining attention for controlling crop pathogens.

The use of natural resources, especially plant species, as therapeutic agents to treat various ailments in both humans and animals has been a global topic for years. An investigation of bioactive secondary metabolites from plants can result in new fungicides that can be utilized to fight crop diseases in the field and during post-harvest storage [21]. Plants' bioactive metabolites can be developed into a new class of fungicides with low toxicity toward non-target organisms [22, 23]. They are also relatively biodegradable, therefore, their residues may not remain in the surroundings for longer [24].

*Lantana camara* L., commonly known as wild or red sage, bunchberry, bird's brandy, cherry pie, or tick-berry, belongs to the family Verbenaceae [25]. It was introduced in many countries as a decorative or ornamental plant [26, 27]. However, it is currently regarded as a notorious invasive species and as an agricultural weed [28]. As an invasive plant, it is known to inhibit and suppress the growth of some native plants [29], leading to a decline in the diversity of invertebrate population and indigenous vegetation in most part of Pietermaritzburg and Cape of Good Hope in South Africa [30]. Moreover, *L. camara* has potential to overgrow pastures and it often increases flammability of the fire-sensitive dry rainforest vegetation [31]. *Lantana camara* is a well-known folk medicinal plant that has been used as remedy for numerous ailments [32]. Its leaves and/or oil from the leaves are used as antiseptic for wounds and swelling [33, 34]. A decoction made from its roots or leaves is used for the relief of toothache, headaches, and to treat cough [33, 34]. Pharmacological studies conducted on its different parts revealed antiproliferative, antioxidant, fungicidal, nematicidal, and insecticidal activities [35, 36, 37, 38, 39]. The present study has been embarked on characterizing the compounds from *L*. *camara* and to investigate their antifungal efficacy, adverse effect on seed germination, and toxicity towards macrophage cells. Antifungal evaluation of compounds isolated from this plant against *Fusarium* species may offer foundational information for the development and application of bio-fungicides in crop protection, while creating a beneficial use in the control of this invasive plant. This is the first report on antifungal efficiency of lantadene A and boswellic acid separated from the leafy part of *L*. *camara* against phytopathogenic *Fusarium* species.

## 2 Materials and methods

### 2.1 Plant material

The leaves of *L*. *camara* were collected during spring season at Roodeplaat (S 25°36.206′, E 028°20.915′). An herbarium voucher specimen (Voucher number UNIN 121003) was deposited at the University of Limpopo.

### 2.2 Preparation of extracts

Green fresh leaves (10 kg) of *L*. *camara* were shade-dried at ambient temperature and then grounded using a milling machine. The material was extracted (300 g powder/3.0 L solvent) with ethyl acetate solvent on a shaker for an hour. The extract was filtered and the residual plant material was re-extracted. The extract was concentrated at a temperature below 45 °C using a Stuart rotary evaporator. Thereafter, it was air-dried in a fume hood and kept in an airtight container.

### 2.3 Fusarium fungal strains

*Fusarium subglutinans* (PPRI 6740), *F. proliferatum* (PPRI 18679), *F. solani* (PPRI 19147), *F. graminearum* (PPRI 10728), and *F. semitectum* (PPRI 6739) were obtained from the Agricultural Research Council – Plant Health and Protection, Pretoria, South Africa. The fungal strains were sub-cultured as described in the previous study [40]. Each pathogen was sub-cultured on Potato Dextrose Agar (Merck, South Africa) and allowed to grow for three to four days at suitable condition (27 °C). After this period, the fungal mycelia were scrapped off (1% inoculum) and introduced in the broth (Merck, South Africa), which was incubated further for three days. Fungal spores were collected by straining cultured broth through a double-layer cheesecloth and the number of fungal spores was determined using inverted microscope and haemocytometer. The spores were adjusted to a final concentration of 1.0 × 10^6^ spores/mL Potato Dextrose Broth before the antifungal activity assay [40, 41, 42].

### 2.4 Isolation of compounds

Ethyl acetate extract (3.5 g) was dissolved in 20 mL acetone and mixed with 15 g silica gel. The silica gel-extract mixture was dried in a fume hood and crushed into a fine powder using a mortar and pestle. A silica gel column was used to separate and purify the fractions as outlined by Seepe et al. [43]. The column was eluted with 100% petroleum ether as solvent and thereafter, 50 mL of mobile phase mixtures (v/v): petroleum ether: ethyl acetate and ethyl acetate: methanol at different solvent ratio. The column yielded thirteen fractions (**L–X**). The fractions were cleaned into pure compounds through thin layer chromatography using toluene: methanol: acetonitrile: acetic acid (80:10:5:5) as eluent. Fractions **L** (153 mg) and **R** (120 mg) were targeted for their evaluation of antifungal activity, cytotoxicity, phytotoxicity, and structural characterization because of the higher yield obtained.

### 2.5 Antifungal efficiency of separated fractions

The activity of separated fractions was evaluated against *Fusarium* strains using a microplate dilution assay [44]. In brief, potato dextrose broth was dispensed in all microplate wells. A hundred microliters of the or fraction at 10 mg/mL were added to the first well and diluted two-fold. The fungal pathogen in broth (100 μL) adjusted to 1.0 × 10^6^ spores/mL was dispensed into each treatment wells. Amphotericin B® (Phytotek Lab Suppliers) was a positive control although acetone, sterile water, and potato dextrose broth were negative controls. The micro-plate was secured and incubated for three days. *p*-Iodonitrotetrazolium chloride (INT) was added to indicate the fungal growth. The (MIC) values were reported as the lowest amount of fractions that suppressed the growth of tested pathogens as evident in no color change after incubation with INT [45]. The antifungal assay was conducted in triplicate and repeated twice.

### 2.6 Effect of separated fractions on seed germination

Phytotoxicity of isolated or separated fractions **L** and **R** on maize seeds was evaluated as detailed by Seepe et al. [40]. Approximately 250 seeds were soaked overnight in fraction **L** and another set in fraction **R**, at fixed concentration (0.63 mg/mL) in aqueous acetone. The selection of 0.63 mg/mL concentration was based on the highest amount of the fractions that demonstrated a MIC value less than 1.0 mg/mL against the screened *Fusarium* strains. The controls used were water and 10% aqueous acetone. The seeds were arranged in the per Petri dish and incubated in a growth chamber at a constant temperature (25 °C) in the alternating light and darkness (12 h). Germinated seeds were recorded three days after incubation and the percentage germination was computed by the following formula:

$$Percentage\;seed\;germination=\left(\frac{Number\;of\;germinated\;seeds}{Total\;number\;of\;seeds}\right)\times 100$$

### 2.7 Cytotoxicity assessment of separated or isolated fractions

The Raw 264.7 cells were cultured in a micro-plate well at a known density known (6×10^4^ per well). It was placed at 37 °C in a CO_2_ incubator for overnight. MTT (3-(4,5-dimethylthiazol-2-yl)-2,5-diphenyl tetrazolium Bromide) assay was used to evaluate cell viability [46]. The cells were treated for 24 h with each fraction at different strength (25, 50, and 100 μg/mL); 0.1% dimethyl sulfoxide and 50 μg/mL curcumin. Dimethyl sulfoxide and curcumin were controls used to validated the experiment or assay. The control macrophage cells were boosted with 2% foetal bovine serum (FBS). The absorbance reading were recorded using microplate reader. The percentage cell viability was calculated. The amount of the fraction needed to suppress cell multiplication or growth by 50% was generated from dose-response curve using GraphPad Prism.

### 2.8 Instrumentation used to characterize isolated fractions

Melting point of fractions **L** and **R** were determined using Stuart SMP3 apparatus. The maximum absorption spectrum was recorded on a spectrophotometer and liquid chromatography-photodiode array detector. Mass spectrophotometric (MS) spectra were recorded on a liquid chromatography-mass spectrometer (LC-MS-2020, Shimadzu, Scientific Instruments, Japan). The instrument has electrospray ionization source recording in the negative (*m*/*z* 250 to 1000) and positive (*m*/*z* 250 to 1000) techniques.

Each fraction was dissolved in HPLC grade acetonitrile (Lab-scan analytical sciences) and approximately 2 μL of 10 mg/mL was injected into the chromatography system equipped with a reverse phase C18 column. The mobile phase consisted of mixture A (10 mM ammonium formate dissolved in 90% acetonitrile: water) and mixture B (0.1% formic acid in acetonitrile, v/v). The LC and UV grade water was used to prepare the mobile phases. Isocratic elution was established with 30% mixture A and 70% mixture B delivered at a flow rate of 200 μL/min. Reserpine and nitrophenol dissolved in HPLC grade acetonitrile were separately used as standards to calibrate the detector (MS). The analysis was done using Lab Solution application software and was documented as absolute intensity against mass to charge ratio (*m*/*z*) values. The mass spectroscopy results were exported to *m*/*z* cloud application software to search for closely related or potential chemical compounds matching the MS fingerprint [47].

Nuclear Magnetic Resonance (NMR) analysis, i.e. ^1^H NMR, ^13^C NMR, and 2 dimensional NMR spectra were achieved using NMR, Ascend 400 MHz Topspin 3.2 spectrometer operating at 400 MHz (^1^H) and 100 MHz (^13^C). The fraction was dissolved in deuterated chloroform (CDCl_3_) in a clean and dried NMR tube. The chemical shifts (δ) were measured in ppm, relative to residual deuterated solvent resonance used as a reference, ^1^H NMR: 7.25 ppm and ^13^C NMR: 77.0 ppm. Multiplicity of signal was abbreviated as follows: s (singlet), d (doublet), dd (doublet of doublet), t (triplet), q (quartet), and m (multiplet). The numbering of the atoms is for convenience only and does not implies the nomenclature numbering of the chemical compounds.

### 2.9 Statistical analysis

Data from phytotoxicity and cytotoxicity experiments were separately treated statistically. The treatments were evaluated using a one-way analysis of variance. Where a statistical significance (p = 0.05) was noted, means separation was computed with Duncan's Multiple Range Test.

## 3 Results

### 3.1 Antifungal efficiency of separated fractions

Of the thirteen fractions obtained from the leafy part of *L*. *camara*, eight fractions demonstrated activity with MIC value less than or equal to 0.63 mg/mL towards all the five tested *Fusarium* strains (Table 1). Fraction **X** exhibited strong antifungal activity (MIC = 0.63 mg/mL) against *F. subglutinans* and *F. proliferatum*, but it was poorly active (MIC = 1.3 mg/mL) towards *F. semitectum*, *F. graminearum*, and *F. solani*. Fraction **L** demonstrated strong activity against all pathogens while fraction **R** showed strong activity (MIC = 0.63 mg/mL) against only two pathogens (*F. subglutinans* and *F. semitectum*). Except for *F. proliferatum*, antifungal activity of fraction **L** against *F. semitectum F. graminearum*, *F. solani*, and *F. subglutinans* was stronger than the activity exhibited by amphotericin B. Fraction **R** demonstrated activity that is stronger than positive control against only *F. subglutinans* and *F. semitectum* (Table 1).

**Table 1:** Minimum inhibitory concentration values of fractions obtained from ethyl acetate leafy extract of *L*. *camara* against *Fusarium* pathogens.

Fraction	MIC value (mg/mL)				
	*F. subglutinans*	*F. proliferatum*	*F. solani*	*F. graminearum*	*F. semitectum*
**L**	**0.63**	**0.04**	**0.63**	**0.63**	**0.63**
**M**	> 2.5	2.5	1.3	> 2.5	2.5
**N**	1.3	**0.63**	**0.63**	1.3	**0.16**
**O**	1.3	**0.16**	**0.63**	**0.63**	**0.63**
**P**	**0.31**	**0.31**	**0.31**	**0.63**	**0.31**
**Q**	**0.63**	**0.63**	**0.63**	**0.63**	**0.31**
**R**	**0.63**	1.3	2.5	2.5	**0.63**
**S**	**0.31**	**0.16**	**0.31**	**0.31**	**0.16**
**T**	**0.31**	**0.16**	**0.31**	**0.63**	**0.31**
**U**	**0.16**	**0.08**	**0.31**	**0.63**	**0.08**
**V**	**0.31**	**0.16**	**0.31**	**0.63**	**0.31**
**W**	**0.63**	**0.63**	**0.31**	**0.63**	**0.31**
**X**	**0.63**	**0.63**	1.3	1.3	1.3
Amphotericin B	9.4	0.0004	1.2	2.3	2.3

The bold values indicate antifungal efficiency with MIC of less than 1.0 mg/mL.

### 3.2 Phytotoxicity effect of isolated fractions **L** and **R** seed germination

The treatments (maize seeds soaked in isolated fraction) and controls (untreated maize seeds) demonstrated germination of 92% on average. Furthermore, there is no statistically significant difference between the treatments and controls (Table 2). There is also no significant difference between the seedling root length or seedling shoot length of treated and untreated maize seeds.

**Table 2:** Effect of fractions **L** and **R** on seed germination. The fractions were reconstituted in 10% aqueous acetone at fixed amount of 0.63 mg/mL.

Treatment	Seed germination (%)	Seedling root length (cm)	Seedling shoot length (cm)
Water	93.68 ± 1.69 a	0.91 ± 0.21 a	0.39 ± 0.08 a
10% aqueous acetone	94.46 ± 0.82 a	0.65 ± 0.11 a	0.32 ± 0.06 ab
Fraction **L**	92.57 ± 1.78 a	0.86 ± 0.09 a	0.35 ± 0.02 ab
Fraction **R**	93.83 ± 1.79 a	0.73 ± 0.12 a	0.37 ± 0.04 a

The experiment consisted of five replicates per treatment and data were averaged and treated statistically. Mean values within the column with the same letters show no significant differences (p = 0.05), as resolved with DMRTest.

### 3.3 Cytotoxicity of fractions **L** and **R** on Raw 264.7 macrophage cells

Both fractions **L** and **R** constrained the growth of Raw 264.7 cells in a dose-dependent fashion. There is no significant difference between the percentage cell viability of positive control (curcumin) and fraction **R** at the same concentration range (Table 3). The half-maximal inhibitory concentration (IC_50_) values were 84.2 μg/mL and 186.6 μg/mL for fractions **L** and **R**, respectively.

**Table 3:** Cytotoxicity of isolated fractions **L** and **R** towards Raw 264.7 cells.

Treatment	Cell viability (%)
Untreated cells	100.00 ± 0.00 d
0.1% DMSO	81.97 ± 2.16 ab
Curcumin 50 μg/mL	80.15 ± 1.90 a
Fraction **L** (25 μg/mL)	86.37 ± 1.02 b
Fraction **L** (50 μg/mL)	67.09 ± 1.49 c
Fraction **L** (100 μg/mL)	44.43 ± 2.12 e
Fraction **R** (25 μg/mL)	95.46 ± 1.52 d
Fraction **R** (50 μg/mL)	84.28 ± 1.14 ab
Fraction **R** (100 μg/mL)	70.40 ± 1.92 c

The assay was replicated twice. Data were averaged and treated statistically. Mean values within the column with the same letters show no significant differences (p = 0.05), as resolved with DMRTest. DMSO = dimethyl sulfoxide.

### 3.4 Structural elucidation

#### 3.4.1 Fraction **L**

Fraction **L** was isolated as a brown-orange powder and has a melting point of 275 °C–285 °C. Its UV absorption maxima were 448 nm in both methanol and ethanol. The mass spectrum (positive ESI) displayed peaks at *m*/*z* 563.20, 507.72, 551.35, 593.20, 639.35, 683.35, 727.45, 905.5, 963.56 and parental peak at *m*/*z* 569 [M + H]^+^, which is consistent with the molecular formula C_35_H_52_O_6_. The spectra (^13^C and ^1^H-NMR) of fraction **L** are compared with published data in Table 4 and Table 5, respectively.

**Table 4:** Comparison of ^13^C-NMR data of fraction **L** with reported literature [48, 49].

Fraction **L**		Literature data (δ_C_ (ppm in CDCl_3_)	
δ_C_ (ppm in CDCl_3_)	DEPT	[48]	[49]
15.8	CH_3_	15.9	15.1
17.2	CH_3_	16.8	16.8
18.3	CH_3_	-	-
19.6	CH_2_	19.3	19.6
20.5	CH_3_	20.8	20.5
23.7	CH_3_	23.9	23.5
24.1	CH_2_	24.3	24.2
25.3	C	-	25.7
26.0	CH_2_	26.3	26.1
27.7	CH_3_	27.9	27.6
29.3	CH_2_	–	–
30.1	CH_2_	30.3	30.0
30.6	CH_2_	–	–
33.7	CH_2_	33.9	33.7
34.5	CH_2_	34.4	34.1
35.0	C	36.7	36.8
38.2	CH_2_	38.6	38.4
39.0	C	39.3	39.2
41.9	CH_2_	42.2	41.9
45.7	CH	46.2	46.9
50.1	CH_2_	50.3	
50.7	C	50.6	50.6
67.7	CH	65.8	–
75.7	C	–	75.8
98.8	C	–	–
115.9	CH	–	–
122.4	CH	122.3	–
127.7	C	127.9	127.6
138.4	CH	138.9	138.9
143.5	C	143.5	143.1
157.2	C	–	–
166.5	C	166.7	166.3
178.9	C	177.7	180.1
182.9	C	221.9	217.7

**Table 5:** ^1^H-NMR data of fraction **L** in deuterated chloroform.

Fraction **L**			Literature compound [48]
δ_H_ (ppm in CDCl_3_)	Multiplicity	Coupling Constants (J, Hz)	δ_H_ (ppm in CDCl_3_)
6.00	1H, q	1.4, 5.8, 1.4	5.98
5.39	2H, s		5.37
5.29	1H, t	13.8	-
5.06	2H, d	5.9	5.09
4.22	1H, t	8.3	-
3.87	2H, d	6.6	3.99
3.04	2H, td	9.4	3.05
2.80	1H, d	10.3	2.38
2.14	3H, s		–
1.97	4H, dd	5.7, 1.5	1.95
1.86	2H, d	21.3	1.88
1.81	6H, s		1.80
1.25	6H s		1.25
1.15	6H, s		–
1.06	2H, t	9.5	–
0.97	9H, d	13.4	0.98
0.85	2H, d	6.5	0.84

An examination of ^13^C NMR and Distortionless Enhancement by Polarization Transfer (DEPT) experiments showed 12 quaternary carbons. These quaternary carbon signals resonated at 25.3, 35.0, 39.0, 50.7, 75.7, 98.8, 127.7, 143.5, 157.2, 166.5, 178.9 and 182.9 ppm. The proton spectrum of fraction **L** showed signals ranging from 0.85 to 6.00 ppm assignable to methyl (–CH_3_), methylene (–CH_2_), and methine (–CH) protons. The protons at 6.00 and 5.29 ppm were at a chemical shift corresponding to the alkene region; therefore, they may either be assigned to protons connected to C-33 or C-12. The Heteronuclear Single-Quantum Correlation (HSQC) experiment showed coupling between proton at 6.00 ppm and carbon signal at 138.4 ppm (C-33), while protons at 5.29 ppm showed direct attachment to carbon signal at 122.4 ppm (C-12). Multiplicity (triplet) of protons at 5.29 ppm may be due to the split coupling of two protons attached to C-11. The proton-proton Correlation Spectroscopy (^1^H–^1^H COSY) data showed split coupling between δ_H_ 5.29 ppm and 1.86 ppm, assuming that these protons (δ_H_ 1.86 ppm) were attached to C-11. The signal 1.86 ppm split coupled and appeared as a doublet, confirming the attachment of one proton atom on the neighboring carbon (C-12). This assignment assumed that C-13 is quaternary; hence, the proton COSY spectrum showed the coupling between H-11 (δ_H_ 1.86 ppm) and H-12 (δ_H_ 5.29 ppm) only. This quaternary C-13 was tentatively assigned to 143.5 ppm since it resonated with the chemical shift characteristics of the alkene region. However, there was another proton signal at the alkene region (6.00 ppm) resonating as multiplet/quartet. This multiplicity may be due to a split coupled with methyl protons at C-34. This assignment was substantiated by HSQC observation, which indicated a coupling between proton (δ_H_ 6.00 ppm) and C-33 (138.4 ppm). Examination of ^13^C NMR and DEPT experiments further confirmed the assignment of carbon signal at 138.4 ppm to C-33.

**Figure 1:**
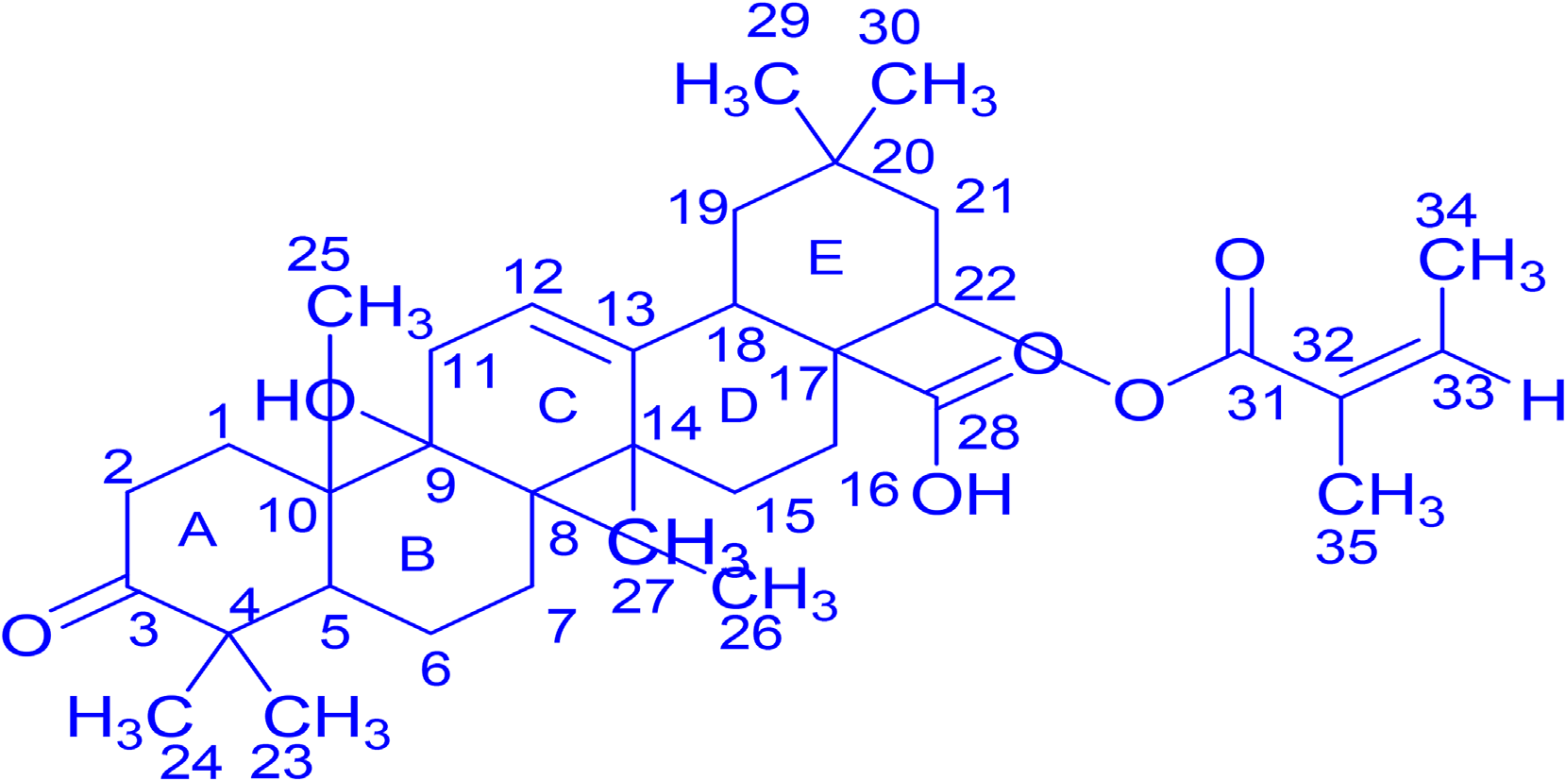
The chemical structure of antifungal isolated as fraction **L** from leaf extract of *Lantana camara*.

Meanwhile, the proton signal resonating at 4.22 ppm was at a chemical shift corresponding to CH attached to oxygen (CH–O–); hence, it was tentatively assigned to the proton connected to C-22. The multiplicity (triplet) of this proton may be due to two protons attached to C-21. HSQC experiment exhibited coupling between this proton (4.22 ppm) and carbon resonating at 67.7 ppm. Another signal in the carbonyl region was quaternary carbon at 182.9 ppm, which matches ketone carbonyl resonance and was assigned to C-3. The other carbonyl peak at 178.9 ppm was characteristic of the carboxylic acid group; therefore, was allocated to C-28. From the DEPT experiment, the signal at 166.5 ppm appeared as quaternary carbon and since it was within the chemical shift characteristics of carbonyl esters, thus was ascribed to C-31. There were a set of six proton signals at 1.15 ppm and 1.25 ppm, which were tentatively assigned to methyl protons attached to C-29, 30, and C-23, 24, respectively. The other six proton signals at 0.97 ppm which split coupled into doublet can be allocated to protons attached to C-34 and C-35. The combination of ^13^C NMR and DEPT experiments made the assignment of methyl and methylene carbons possible as were assignable to chemical shifts ranging from 50.1 to 15.8 ppm. Based on spectroscopic information and published data, fraction **L** was tentatively characterized as lantadene A (22-angeloyloxy-9-hydroxy-3-oxo-olean-12-en-28-oic acid) and its structure is shown in Figure 1. The individual carbon atoms of fraction **L** were assigned to relevant chemical shift signals as shown on ^13^C NMR spectrum in Figure S1.

#### 3.4.2 Fraction **R**

**Figure 2:**
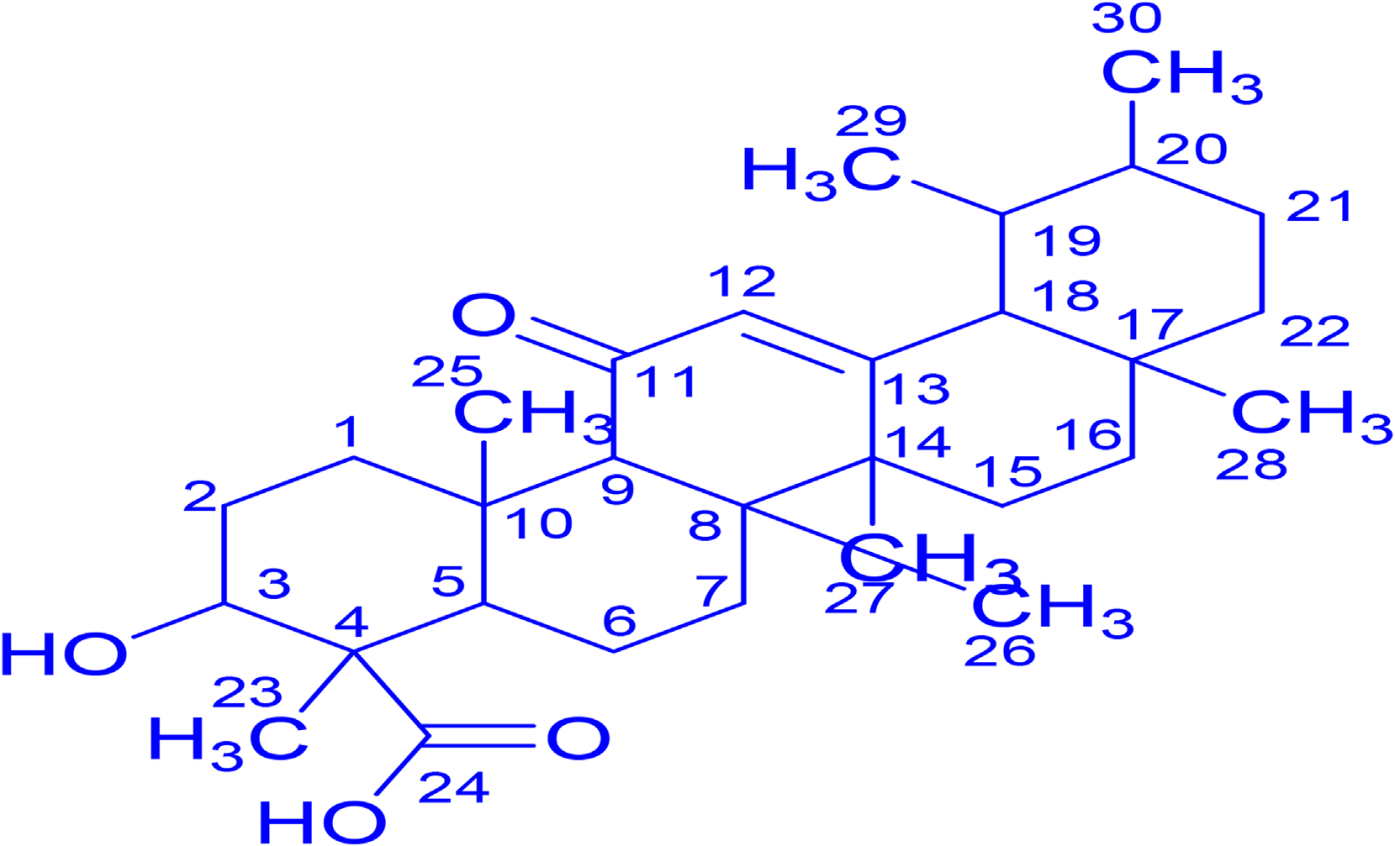
The chemical structure of antifungal isolated as fraction **R** from leaf extract of *Lantana camara*.

Fraction **R** was isolated as a yellow to whitish substance and its recorded melting point was 190 °C–195 °C. The mass spectrum (negative ESI) displayed peaks at *m*/*z* 485.2, 515.3, 555.3, 583.3, 599.3, 939.6, and base peak at 469.3. This peak was consistent with the molecular formula C_30_H_46_O_4_. ^13^C NMR and ^1^H NMR spectral details of fraction **R** are presented in Tables 6 and 7, respectively. The ^13^C NMR and DEPT experiments displayed eight quaternary carbons, seven methyl groups (–CH_3_), seven methylene (–CH_2_), and six methines (–CH) signals. Quaternary signals resonating at δ_C_ 32.2, 35.02, 40.18, 41.99, 48.06, 143.00, 173.00, 182.55 ppm can be allocated to C-17, C-10, C-8, C-14, C- 4, C-13, C-11 and C-24, respectively. Detailed examination of spectroscopic data (MS and NMR) and a melting point of fraction **R** in comparison to data for boswellic aldehyde, 3-O-accetly-11-keto-β-boswellic acid, and 24-norursa-3,12-dien-11-one obtained from literature led to the identification of fraction **R** as 11-keto-β-boswellic acid. The chemical structure of 11-keto-β-boswellic acid is exhibited in Figure 2. The individual carbon atoms of fraction **R** were assigned to relevant chemical shift signals as shown on ^13^C NMR spectrum in Figure S2.

**Table 6:** Comparison of ^13^C NMR data of fraction **R** with reported literature [50, 51, 52].

Fraction **R**		Literature compounds		
δ_C_ (ppm in CDCl_3_)	DEPT	Boswellic aldehyde [50]	3-O-accetly-11-keto-β-boswellic acid [51]	24-norursa-3,12-dien-11-one [52]
15.6	CH_3_	15.6	–	–
16.9		16.6	–	–
17.5	CH_3_	–	17.4	17.7
17.8		18.1	–	–
18.3	CH_3_	18.2	18.3	–
19.6	CH_2_	–	–	19.6
20.5	CH_3_	–	20.5	20.3
21.1	CH_3_	21.2	21.2	20.9
23.1	CH_3_	22.0	23.5	22.2
23.7	CH_3_	–	23.8	23.6
25.3	CH	26.7	–	–
27.2	CH_2_	27.2	27.2	27.7
29.3	CH_2_	28.1	28.9	29.2
30.9	C	–	30.9	–
31.2	C	31.1	32.8	31.5
34.8	CH_2_	33.6	34.6	34.2
35.0	C	35.5	37.3	35.6
38.8	CH_2_	–	–	–
39.1	CH_2_	39.3	39.3	39.5
40.2	CH	40.0	40.9	39.6
41.7	C	41.3	–	41.4
42.0	CH	42.0	43.7	45.3
48.1		49.0	46.4	49.4
50.1	CH	–	50.4	–
53.1	C	55.7	–	–
67.8	CH_2_	–	–	–
75.0	CH	–	73.0	-
122.5		–	–	122.5
125.6	CH	124.5	–	–
138.1		–	130.5	134.1
143.0	C	146.3	–	–
173.0	C	177.5	170.2	163.2
182.6	C	–	–	198.6

**Table 7:** Proton NMR data of fraction **R** in deuterated chloroform.

Chemical Shift (H, ppm in CDCl_3_)	Multiplicity	Coupling Constants (J, Hz)
5.99	m	5.6
5.56	s	
5.36	s	
5.24	d	14.4
5.04	d	16.4
4.23	t	8.0
3.88	d	8.8
3.06	m	10.0
2.81	dd	44, 13.6
2.34	s	
2.12	m	
1.78	t	1.6
1.68	s	
1.67	m	
1.49	s	
1.48	s	
1.23	m	
1.13	d	12.0
1.01	m	
1.00	m	
0.94	m	
0.87	d	7.2
0.74	d	14.4
0.70	s

## 4 Discussion

Evaluation of antifungal efficiency of separated fractions is important for the selection of potent fractions which may be purified further to determine their chemical structures. The selection of such fractions for further studies depends largely on their activity (MIC value ≤ 1.0 mg/mL), the number of phytopathogens inhibited, and the quantity of purified material. It was noticed that the antifungal efficiency of the fractions is pathogen-specific. As an example, fraction **R** was active (MIC = 0.63 mg/mL) against *F. subglutinans* and *F. semitectum*, meanwhile, it was poorly active (MIC ≥1.3, 2.5, and 2.5 mg/mL) against *F. proliferatum*, *F. solani*, and *F*. *gramineraum*, respectively. Plant extracts exhibiting a MIC value of less than 1.0 mg/mL are considered to have a strong or good activity [53], while extracts with a MIC value of less than 0.1 mg/mL are classified as having a very good antimicrobial activity [54, 55]. Isolated phytochemicals demonstrating a MIC value of less than 1.0 mg/mL are considered as having a very active antimicrobial activity [56]. The fractions **L** and **R** exhibited stronger activity than amphotericin B against many of the evaluated pathogens. Based on literature criteria and comparison with MIC value reported for standard antifungal compound (amphotericin B) used in the present investigation, many of the separated fractions may be classified generally as having good or strong antifungal activity. In our previous study, a crude ethyl acetate extract from the leafy part of *L*. *camara* displayed strong antifungal efficiency with MIC values of 0.04 mg/mL and 0.08 mg/mL against *F. subglutinans* and *F. semitectum*, respectively [57]. The discrepancy between the activity of fraction **R** and the crude extract against the same pathogens may be due to the synergistic effect of various chemical constituents in the crude extract. Mdee et al. [58] also reported that *L*. *camara* extract exhibited strong antifungal activity (MIC value = 0.08 mg/mL) against *F. oxysporum*, a soil-borne fungal pathogen that is also well-known to cause various crop diseases. A positive control was evaluated at the same concentration as the isolated fractions and was used to validate the assays in our study. Plant extracts, fractions or isolated compounds exhibiting stronger or comparable activity to positive control are of particular interest in the light of an urgent need to discover new antimicrobial compounds to manage the problem of microbial resistance [59]. Despite the good activity exhibited by other fractions, fractions **L** and **R** were selected for further studies due to their higher quantity and purity compared to other fractions.

Fractions **L** and **R** showed no harmful effect on seed germination and initial seedling growth when compared to water treatment used as the control. A recent study showed that *L*. *camara* extract has the potential to protect stored maize seeds against *Sitophilus zeamais* and *Prostephanus truncates*, and it also resulted in a higher maize seed germination (94%) as opposed to negative control (82%) [60]. In that study, no attempt was made to isolate the active compounds, however, the authors suggested that the observed activity might be as a result of alkaloids, tannins, terpenes, and steroids [60]. Postharvest losses of maize seeds due to *Fusarium* pathogens and other microorganisms is an important constraint factor in maize production. In resource-poor farming, excess maize seeds are reserved for forthcoming planting season [61]. To discover plant-based pesticides for protecting maize seeds during storage, it is also important to evaluate the effect of such products on seed germination.

Moreover, stored maize seeds are also consumed throughout the year in resource-poor communities. This calls for the use of natural products, which not only protect maize seeds from spoilage against *Fusarium* pathogens but also are less harmful to humans. There are several reports on the toxicity of *L*. *camara* on livestock, nonetheless, that resulted from consumption of a higher quantity of plant material [27, 62, 63]. Tokarnia et al. [64] reported that lethal poisoning from *L*. *camara* admitted as a single dose of 40 g/kg in cattle. The leaves (340–453 g) of the plant are believed to cause liver, gall bladder, and kidney damage in horses, cattle, and sheep [65]. The toxicity principle of *L*. *camara* had been associated with pentacyclic triterpenes compounds such as lantadene A and B [66, 67, 68].

In a study conducted by Heikel et al. [69], lantadene A was reported to be icterogenic to the rabbit. However, three years later it was found that chromatographically pure lantadene A does not show hepatotoxicity in rabbits [70]. Another study demonstrated that lantadene A is non-icterogenic and nontoxic to lambs and guinea pigs [71, 72]. Carstairs et al. [73], concluded that ingestion of any part of *L*. *camara* does not cause significant toxicity in humans. *Lantana camara* leaves are boiled for tea and humans use the decoction as an effective remedy for cough [25].

More clinical studies are required to evaluate the toxicity of fractions and pure compounds isolated from different parts of *L. camara*. In our study, both fractions **L** and **R** from *L. camara* leaves showed no severe totoxicity towards Raw 264.7 macrophage cells when matched to curcumin (positive control). The cytotoxicity of both fractions (**L** and **R**) was dose-dependent with recorded IC_50_ values of 84.2 μg/mL and 186.6 μg/mL, respectively. The National Cancer Institute (NCI) in USA, classifies crude extracts and pure compounds as cytotoxic agents when they indicated IC_50_ values of less than 20 μg/mL and 4 μg/mL, respectively [74].

Spectroscopic characterization of fractions **L** and **R** led to lantadene A (22-angeloyloxy-9-hydroxy-3-oxo-olean-12-en-28-oic acid) and boswellic acid (11-keto-β-boswellic acid), respectively. Lantadene A is a pentacyclic triterpene present in the leaves of *Lantana* species [67, 75, 76]. Lantadene A obtained from leafy part of *L*. *camara* was reported to induce apoptosis in human leukemia HL-60 cells [77]. It was also effective in inhibiting the growth of LNCap cells (human prostate adenocarcinoma cells) without causing any cytotoxic effect on the RWPE-1 cells [78]. This compound is also a potential lead molecule for the development of treatment for onchocerciasis; a chronic nematode-borne disease affecting the skin and eyes [79]. To our understanding, there is a shortage of information on the activity of lantadene A isolated from the leaves of *L*. *camara* against phytopathogens.

The 11-keto-β-boswellic acid is a major boswellic acid mostly isolated from the gum resin of various *Boswellia* species belonging to the Burseraceae family [80, 81, 82]. The present study for the first time isolated 11-keto-β-boswellic acid from leafy part of *L*. *camara* and demonstrated its efficiency against *Fusarium* pathogens. This compound was described to reveal antibacterial activity with a MIC value of less than 32 μg/mL against vancomycin-resistant *E. faecalis* MRSA ATCC 3591, *S*. *epidermidis* ATCC 12228, *S. aureus* ATCC-29213, *S. mutans* ATCC 25175, *S. sanguinis* ATCC 10556, and *A. viscous* ATCC 15987 [82,83]. Weckesser et al. [84], reported that no antifungal activity was observed when pure 11-keto-β-boswellic acid (128 mg/mL) was evaluated using the agar dilution method against *Candida albicans* and *Candida krusei*. Nevertheless, in the present invistigation compound **R** isolated from *L*. *camara* and characterized as 11-keto-β-boswellic exhibited good activity with MIC value of 0.63 mg/mL against *F. subglutinans* and *F. semitectum*; when using microplate dilution method. Different boswellic acids including 11-keto-β-boswellic acid have been reported to demonstrate anti-inflammatory and anticancer activities [85, 86, 87].

## 5 Conclusions

The current study presented separation and identification of two compounds from the leafy part of *L*. *camara*. Both isolated compounds [lantadene A (22-angeloyloxy-9-hydroxy-3-oxo-olean-12-en-28-oic acid) and boswellic acid (11-keto-β-boswellic acid)] demonstrated strong antifungal activity against some tested *Fusarium* species. When tested against *F. subglutinans* and *F. semitectum*, these compounds exhibited stronger antifungal activity (MIC less than 1.0 mg/ml) compared to the fungicide (Amphotericin B). Both isolated compounds can inhibit the growth of five *Fusarium* pathogens (*F. subglutinans*, *F. proliferatum*, *F. solani*, *F. graminerum* and *F. semitectum*) known to cause massive yield loss in crop production. Furthermore, they showed no cytotoxicity towards Raw 264.7 cells, phytotoxicity against seed germination and seedling growth. Lantadene A and boswellic acid identified in this study can be used as scaffold molecules during the industrial synthesis of bio-pesticide products. This study showed that a notorious weed and an invasive species, *L*. *camara*, may be exploited further into value-added products that can be used as bio-pesticides in crop protection.

## Declarations

### Author contribution statement

Hlabana Alfred Seepe: Conceived and designed the experiments; Performed the experiments; Wrote the paper.

Lerato Raphoko: Performed the experiments; Analyzed and interpreted the data; Contributed reagents, materials, analysis tools or data.

Stephen O. Amoo: Conceived and designed the experiments.

Winston Nxumalo: Conceived and designed the experiments; Analyzed and interpreted the data; Contributed reagents, materials, analysis tools or data.

### Funding statement

This work was funded and supported by the 10.13039/501100001321National Research Foundation, South Africa 544 (NRF Grant No. 98670 and 129370) and the 10.13039/100007537Agricultural Research Council.

### Data availability statement

Data will be made available on request.

### Declaration of interest's statement

The authors declare no conflict of interest.

### Additional information

Supplementary content related to this article has been published online at https://doi.org/10.1016/j.heliyon.2022.e12216.

## Appendix A Supplementary data

Supplementary Materials.docx Supplementary Materials.docx
