# Supplementary material for: Lantadene A and boswellic acid isolated from the leaves of Lantana camara L. have the potential to control phytopathogenic Fusarium species
Source: Heliyon. 2022 Dec 15;8(12):e12216. doi: 10.1016/j.heliyon.2022.e12216 (PMC9792760; doi:10.1016/j.heliyon.2022.e12216)
Supplement: Supplementary Materials.docx [file mmc1.docx]

Article

Lantadene A and boswellic acid isolated from the leaves of *Lantana camara* L. have the potential to control phytopathogenic *Fusarium* species

Hlabana Alfred Seepe ^a,b,c^ *, Lerato Raphoko^b^, Stephen O. Amoo ^a,d,e^, Winston Nxumalo ^b^

^a^ Agricultural Research Council—Vegetables, Industrial and Medicinal Plants, Roodeplaat, Private Bag X293, Pretoria 0001, South Africa

^b^ Department of Chemistry, University of Limpopo, Private Bag X1106, Sovenga, 0727, Polokwane, South Africa

^c^ Döhne Agricultural Development Institute, Plant and Crops Production Research, Private Bag X 15, Stutterheim, 4930, South Africa

^d^ Indigenous Knowledge Systems Centre, Faculty of Natural and Agricultural Sciences, North-West University, Private Bag X2046, Mmabatho 2735, South Africa

^e^ Department of Botany and Plant Biotechnology, Faculty of Science, University of Johannesburg, P.O. Box 524, Auckland Park 2006, South Africa

***** Correspondence: Alfred.Seepe@drdar.gov.za; Tel.: +27 43 683 5455 (H.A. Seepe)

**Supplementary Information**

**1**

**20**

**10**

**17**

**9**

**25**

**26**

**6**

**34**

**16**

**23**

**15**

**29**

**2**

**4**

**14**

**8**

**22**

**12**

**32**

**33**

**13**

**31**

**3**

**28**

**Figure S1**. The ^13^C-NMR spectrum of pentacyclic triterpenoid (fraction **L**) isolated from *Lantana camara* leafy extract.

**10**

**25**

**6**

**16**

**15**

**21**

**19**

**14**

**8**

**4**

**5**

**9**

**3**

**12**

**13**

**11**

**24**

**Figure S2**. The ^13^C-NMR spectrum of pentacyclic triterpene (fraction **R**) isolated from *Lantana camara* leafy extract.
